# Genome-wide identification of WRKY gene family and expression analysis of key *WRKY* genes in response to *Fusarium solani* infection in *Lycium barbarum*

**DOI:** 10.3389/fpls.2025.1543373

**Published:** 2025-05-19

**Authors:** Xia Wu, Nan Li, Bin Wang, Wei Chen, Chongqing Zhang, Yuyan Sun, Jing He

**Affiliations:** ^1^ College of Forestry, Gansu Agricultural University, Lanzhou, China; ^2^ Wolfberry Harmless Cultivation Engineering Research Center of Gansu Province, Lanzhou, China

**Keywords:** *Lycium barbarum*, WRKY gene family, *Fusarium solani*, flavonoid and phenylpropanoid synthesis, expression analysis

## Abstract

Wolfberry (*Lycium barbarum*), a member of the Solanaceae family, is recognized as a pioneering tree species for afforestation in saline-alkali soils and holds significant economic value as a forest species. Its fruit is abundant in bioactive compounds that contribute in both ecological health and human well-being. The WRKY gene family has been extensively studied across various species, with its members’ functions increasingly elucidated. However, limited research has focused on the role of the *WRKY* genes of *L. barbarum*, particularly in resistance to root rot. This study identified the bioinformatics of 104 *WRKY* genes in wolfberry, encompassing phylogenetics, conserved motifs, gene structures, synteny, and collinearity. Based on structural and phylogenetic, the 104 *LbWRKYs* are divided into three main groups, Group I, II and III, with 26, 62 and 15 members, respectively. Synteny analysis revealed high homology between LbWRKY and tomato SlWRKY, with a total of 117 pairs of homologous genes identified. Cis-acting elements analysis demonstrated that subgroup II *LbWRKY* genes contained a higher number of plant hormone-related regulatory elements. Furthermore, 28 *LbWRKY* genes were found to respond to the infection of *Fusarium solani*. Protein-protein interaction prediction and correlation analyses revealed that associations between *LbWRKY* genes and flavonoid and phenylpropanoid synthesis-related genes, and the results showed that *LbWRKY8/100/63/84/102/42/45* was involved in the mechanism of root rot resistance. Expression analysis following *Fusarium solani* inoculation confirmed that these genes participate in root rot resistance in *L. barbarum*. This study provides valuable insights into the functional roles of *LbWRKY* genes, and establishing a foundation for future research on their involvement in secondary metabolite synthesis and their role in enhancing the disease resistance of *L. barbarum*.

## Introduction

Wolfberry *Lycium barbarum* L., a perennial shrub of the Solanaceae family, is valuable medicinal and edible plant (Hu et al., 2014). It is primarily distributed in arid to semi-arid regions of the temperate zones, and its fruit is widely utilized in the pharmaceutical and health care industries (Levin and Miller, 2005; Ma R et al., 2023). Due to its saline-alkaline and drought tolerance, along with its high economic value, wolfberry has become a widely cultivated fruit crop in Northwest China (Yao et al., 2017; Cao et al., 2021; Gong et al., 2022). However, the increasing acreage is accompanied by frequent diseases affecting wolfberry. The root rot, caused by *Fusarium* species, is a common soil-borne fungal disease that poses a serious threat to wolfberry production, leading to reduced yields and compromised crop health (Cichy et al., 2007; Eliane, 2017). Breeding resistant varieties is considered the most effective approach to controlling plant diseases (Agbowuro et al., 2020). Therefore, identifying key disease resistance genes is critical for developing wolfberry varieties resistant to root rot.

Transcription factors (TFs) are regulatory proteins that bind to specific cis-elements in their target genes and modulate the expression level of genes under particular stress conditions. These “cis-trans” interactions play a crucial role in enhancing plant survival under adverse environmental conditions (Shrestha et al., 2018). Several TF families have been identified as key regulators of plant adaptation to stress, including WRKY, bHLH, AP2/ERF, ARF, NAC, MYB, bZIP, among others (Xiao et al., 2023; Ahmad et al., 2024; Bian et al., 2024; Li et al., 2024; Ou et al., 2024; Wang et al., 2024b; Ye et al., 2024). The WRKY family, recognized as one of the most critical transcription factor families, is extensively involved in plant responses to biotic, abiotic and hormonal stresses (Ahuja et al., 2010; Chen et al., 2017; Jiang et al., 2017). For instance, 103 *CpWRKYs* in *Cucurbita pepo* L. are implicated in responses to cold and drought stresses (Liu et al., 2024). Members of the wheat WRKY gene family play a crucial role in the tolerance mechanisms to drought and osmotic stress (Gahlaut et al., 2016; Wani et al., 2018). Similarly, 102 *MeWRKYs* identified in *Manihot esculenta* confer resistance to *Xanthomonas axonopodis* pv. *Manihotis* (Zhu et al., 2022b). Moreover, 63 *AtWRKYs* in *Akebia trifoliata* have been reported to play a significant role in plant disease resistance (Zhu et al., 2022a).

The N-terminus of WRKY DNA-binding domain (DBD) contains a highly conserved sequence, with WRKYGQK as the core motif. Mutants with amino acid substitutions and the featuring either a C-terminus C_2_H_2_ or C_2_HC zinc-binging motif have also been reported (Rushton et al., 2010; Song et al., 2018). Based on the number of WRKY domains and the type of zinc finger motif, WRKY TFs are categorized into three groups, with Group II are further subdivided into five subgroups (IIa–IIe) (Kazuhiko et al., 2005). WRKY TF are involved in phytohormone-mediated signaling pathways, the regulation of pathogenesis-related proteins, and biosynthesis of secondary metabolites by acting on downstream target genes in response to biotic stresses (Li et al., 2020; Nisha and Paramjit, 2021; Luan et al., 2023). In plant, the phenylpropanoid pathway participates in the biosynthesis of secondary metabolites related to disease defense (Zhang et al., 2021). Through the combined analysis of transcriptomic and metabolomic data, *LbPAL*, *LbCHS* and *LbUGT* involved in the biosynthesis of flavonoids and polysaccharides, and their expression was significantly upregulated in response to pathogen infection (Zhang et al., 2021).

*Lycium barbarum* is an important traditional medicinal and food supplement in China, and its genome has recently been released (Cao et al., 2021). Based on this genomic resource, several gene families in *L. barbarum* have been identified, and their biological functions partially elucidated. For instance, 28 *LbaBBXs* and 137 *LbaR2R3MYBs* were identified, with specific members such as *LbaBBX2* and *LbaBBX4*, (*Lba11g0183* and *Lba02g01219*) shown to play regulatory roles in the carotenoids biosynthesis (Yin et al., 2022a, b). Additionally, 12 *LbCCOs* (carotenoid cleavage oxygenase genes) were identified as being involved in regulation of phytohormones, pigments, and aromatic substances (Li et al., 2023). He et al. revealed that 38 *LbAQPs* (aquaporins) are involved in developmental processes and responses to abiotic stress (He et al., 2022). In Solanaceae, the overexpression or silencing of WRKY genes significantly impacts plant defense. For instance, *SlWRKY12* contributes to tomato resistance against *Botrytis cinerea* by positively regulating related defense genes (Liu et al., 2014). Recent studies have demonstrated that the regulation of *CaWRKY40* in pepper and its associated resistance to *Ralstonia solanacearum* depend on the transcriptional activation of CaWRKY06 (Cai et al., 2015). However, comprehensive studies on *WRKY* genes in wolfberry have not yet been reported.

In the current study, we conducted a comprehensive genome-wide identification and analysis of the *WRKY* gene family in *L. barbarum*. We systematically examined their chromosomal location, classifications, conserved protein domains, motif composition, gene structures, phylogenetic relationships, and duplication events. Additionally, we investigated the expression profile of *WRKY* genes in response to inoculation with *F. solani* and explored their potential roles in flavonoid and phenylpropanoid synthesis. These findings identify potential candidate genes for further functional studies and enhance our understanding of the molecular mechanisms underlying lignin synthesis in plants.

## Materials and methods

### Identification and characterization of *LbWRKY* genes in the *L. barbarum* genome

Genome and gene annotation files of *L. barbarum* were downloaded from the NCBI datebase https://www.ncbi.nlm.nih.gov/, the HMMs (PF03106) of the WRKY TFs downloaded from the pfam database http://pfam.xfam.org/ were utilized as a blast search query for WRKY using a threshold E-value of 1e−5. The candidate genes were then further validated using the SMART http://smart.embl-heidelberg.de/ and NCBI-CDD https://www.ncbi.nlm.nih.gov/Structure/cdd/wrpsb.cgi (Lu et al., 2020). Arabidopsis WRKY protein sequences were downloaded from TAIR https://www.arabidopsis.org/, *Solanum lycopersicum* (Sl) ITAG5.0 and *Capsicum annuum* (Ca) were downloaded from Phytozome13 https://phytozome-next.jgi.doe.gov/ and NCBI datebase https://www.ncbi.nlm.nih.gov/. The protein sequences of LbWRKY members were analyzed with Multiple Protein Profiler 1.0 (MPP) https://mproteinprofiler.microbiologyandimmunology.dal.ca/ Predict the physicochemical properties of the proteins and visualize (Sganzerla et al., 2024). The subcellular localization of these proteins was predicted and analyzed using the Plant-mPLoc 2.0 http://www.csbio.sjtu.edu.cn/bioinf/plant/ (Chou and Shen, 2008). TBtools was used to visualize the position of the candidate *WRKY* genes on the chromosome (Chen et al., 2020b).

### Multiple sequence alignment, phylogenetic analysis, gene structure and conserved motif analyze of the WRKY genes in *L. bararum*

The *LbWRKYs* protein sequences were aligned using ClustalW in MEGA7.0, and analyzed the conserved WRKY core domain (60 amino acids) using DNAMAN. The phylogenetic tree of wolfberry and *Arabidopsis thaliana* (*At*) was constructed using the Maximum Composite Likelihood (MCL) model of neighbor-joining (NJ) algorithm in MEGA 7.0 software, and 1,000 iterations of bootstrapping were performed (Chen et al., 2020b). The Evolview 3.0 https://www.evolgenius.info/evolview/#/treeview was used to further modify and enrich the evolutionary tree (Subramanian et al., 2019).

The *WRKY* protein sequences and Genome file of wolfberry were sent to the Visualize Gene Structure in TBtools software to investigate exon-intron structures (Chen et al., 2020b). The conserved motifs of the *LbWRKY* protein sequences were analyzed using MEME https://meme-suite.org/meme/tools/ meme online website with a number of motifs of 10 and optimum width of 6 to 50 bp (Bailey et al., 2009).

### Gene duplication, collinearity analyses and cis-acting elements in the promoters of *LbWRKY* family

The collinearity of *WRKYs* within *L. barbarum* was analyzed by Circos and TBtools software, and the synteny maps of WRKYs between *L. barbarum*, *A. thaliana*, *S. lycopersium* and *C. annuum* were depicted through MCscanX in TBtools (Krzywinski et al., 2009; Chen et al., 2020a). The Adobe Illustrator 2021 software was used to further modify and enrich.

The 2000 bp gene sequence upstream of the initiation codon (ATG) of *LbWRKYs* was identified as the gene promoter sequence. Cis-acting regulatory elements of *LbWRKYs* regions were searched for within 2000bp upstream using PlantCARE tool and then visualized with TBtools (Lescot et al., 2002; Chen et al., 2020b).

### Expression profile of *LbWRKY* family in different developmental stages of *Lycium barbarum* and prediction of miRNA target and SSR

The expression profiles of the LbWRKY gene family in different developmental stages of *L. barbarum* were analyzed using the Genevestigator platform (Hruz et al., 2008). Transcriptome data were obtained from the RefSeq: NCBI reference Sequence Database, and further analyzed using TBtools software. The 3D structure models of key LbWRKY family members are predicted by AlphaFold Protein Structure Database https://alphafold.ebi.ac.uk/ (Jumper et al., 2021). MiRanda software and Trf software were used to predict miRNA and SSR respectively (Enright et al., 2003; Saliminejad et al., 2019). Tomato miRNA was downloaded from plant microRNA database http://bioinformatics.cau.edu.cn/PMRD/ (Zhang et al., 2009).

### RNA-seq analysis

The plant materials used in this study were annual seedlings of Ningqi 7 and provided by Wolfberry producting areas in Baiyin City, Gansu Province. The pathogenic was *Fusarium solani*, which is preserved by the Wolfberry Harmless Cultivation Engineering Research Center of Gansu Province, Lanzhou, China. The leaves of *L. bararum* were collected at 30 d post inoculation (dpi), and stored at -80°C after freezing in liquid nitrogen. The RNA-seq in this study was commissioned by Beijing Biomarker Technologies Co., LTD to complete the library construction, and the cDNA library was sequenced using the Illumina NovaSeq high-throughput sequencing platform.

### Correlation analysis of DEGs of disease resistance and regulatory network of LbWRKYs

The differential genes DEGs (|Log2FC| >1.5, FDR < 0.05) in response to the infection of *F. solani* were analyzed in the transcriptome database, and Pearson correlation analysis in Origin 2022 was used to detect the relationship between resistance-related genes. *Solanum lycopersicum* was used as the reference sequence for string http://string-db.org/ prediction network interaction maps (Szklarczyk et al., 2023). Network diagram was visualized using Cytoscape v3.9.1 (Shannon et al., 2003).

### RNA extraction and quantitative RT-PCR

RNA was extracted from leaves of *L. barbarum* using a Plant RNA Kit (Omega Bio-Tek, Guangzhou, China). PrimeScriptTM RT reagent Kit and TB Green^®^ Premix Ex TaqTM II were used for gDNA erasure and quantitative RT-PCR (Shanghai BioScience Co., Ltd). The primers were designed using NCBI online website **Supplementary Table S9** https://www.ncbi.nlm.nih.gov/tools/primer-blast/index.cgi. For each reaction, three independent biological and technical replicates were used. The relative expression of each gene for RT-qPCR analysis was calculated by 2^−ΔΔCt^ method, Origin 2022 was used for statistical analysis.

## Results

### Identification and characteristics of *LbWRKY* genes

**Figure 1:**
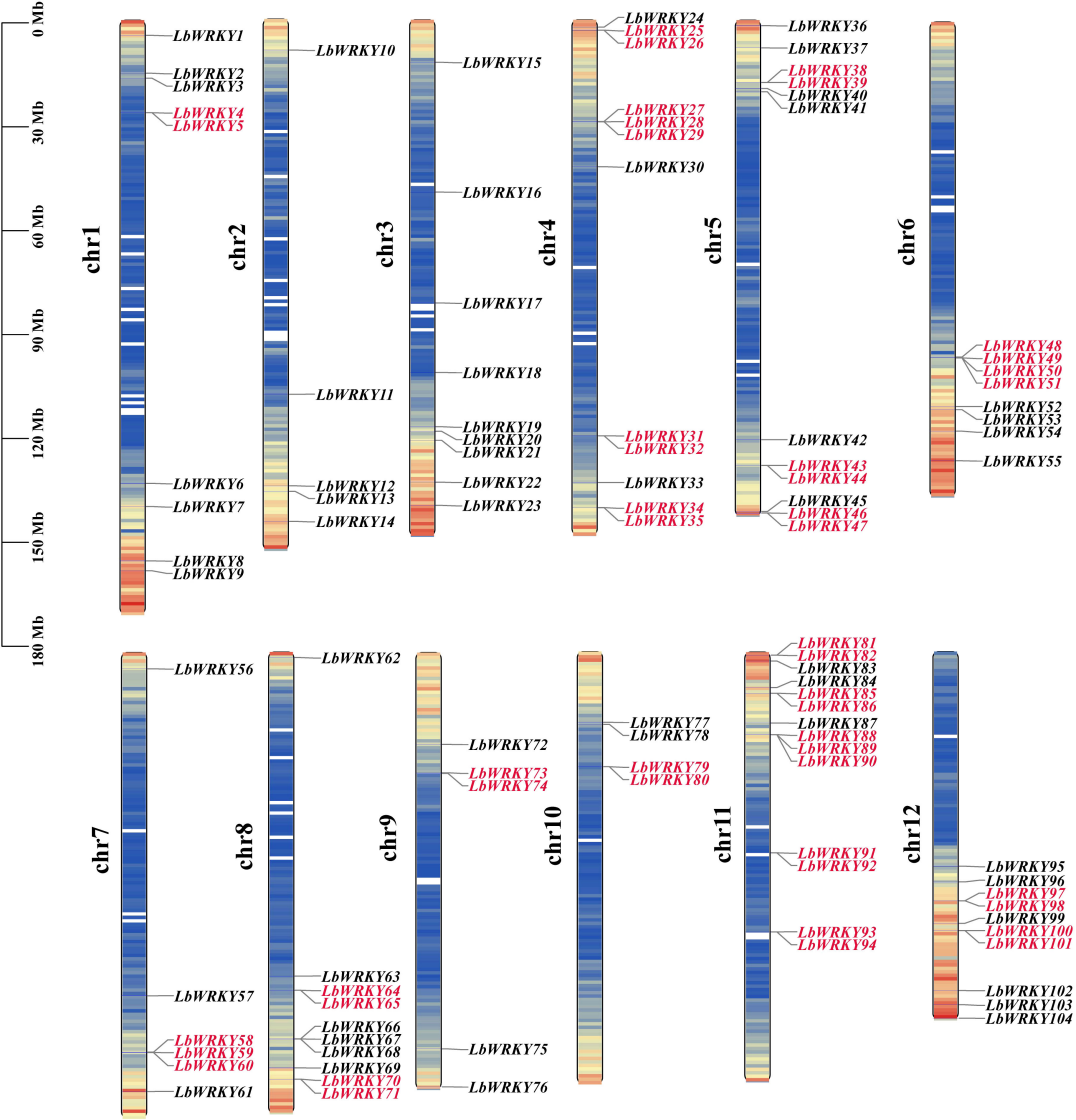
Chromosomal location of *LbWRKY* genes in Wolfberry. At the left of the chromosome is the number of that chromosome. On the right side of every chromosome are the names of the genes. Different colors on the chromosome indicated varying gene densities, with red highlighting the tandem repeat genes.

The WRKY domain HMM profile PF03106 and BLSATP analysis using 71 WRKY Protein sequences from *Arabidopsis thaliana* were employed to identify *WRKY* genes in *L. barbarum* genome. A total of 104 putative *LbWRKY* genes were identified (**Supplementary Table S1**), and named *LbWRKY1* to *LbWRKY104* based on their chromosomal locations. These genes were unevenly distributed across twelve chromosomes (**Figure 1**), with most localized at the ends of chromosomes. Chromosome 11 contained the highest number of *LbWRKYs* genes (13.5%), followed by chromosomes 4 and 5, which each harbored 12 genes, (11.5%). Chromosomes 8 and 12 housed 10 *LbWRKYs* each, while chromosome 10 had the fewest, with only 4 genes (3.8%). The remaining chromosomes 1, 3, 7, 2 and 9 contained separately 9, 9, 6, 5 and 5 *LbWRKY* genes, respectively. Additionally, 47 *LbWRKY* genes, such as *LbWRKY4/5*, *LbWRKY25/26*, *LbWRKY27/28/29*, etc. were clustered into 22 tandam duplication regions on chromosome 1 and 4-12, suggesting potential duplication events.

We analyzed and visualized various physiochemical properties of 104 LbWRKY proteins with MPP profiler (**Supplementary Table S1** and **Supplementary Figure S1**). The amino acid lengths of LbWRKY proteins ranged from 165 (*LbWRKY30*) to 729 (*LbWRKY34*), with predicted molecular weights ranging from 18.96 to 79.73 kDa. Most LbWRKY proteins were acidic, with a pI ratio of approximately 2:1. The GRAVY (grand average of hydropathicity) values for all LbWRKY proteins were negative, indicating there hydrophilic nature. Instability index analysis showed that most of these proteins were unstable. Subcellular localization results revealed that all LbWRKY proteins were located in the nucleus.

### Protein domains and phylogenetic analysis of LbWRKY proteins

**Figure 2:**
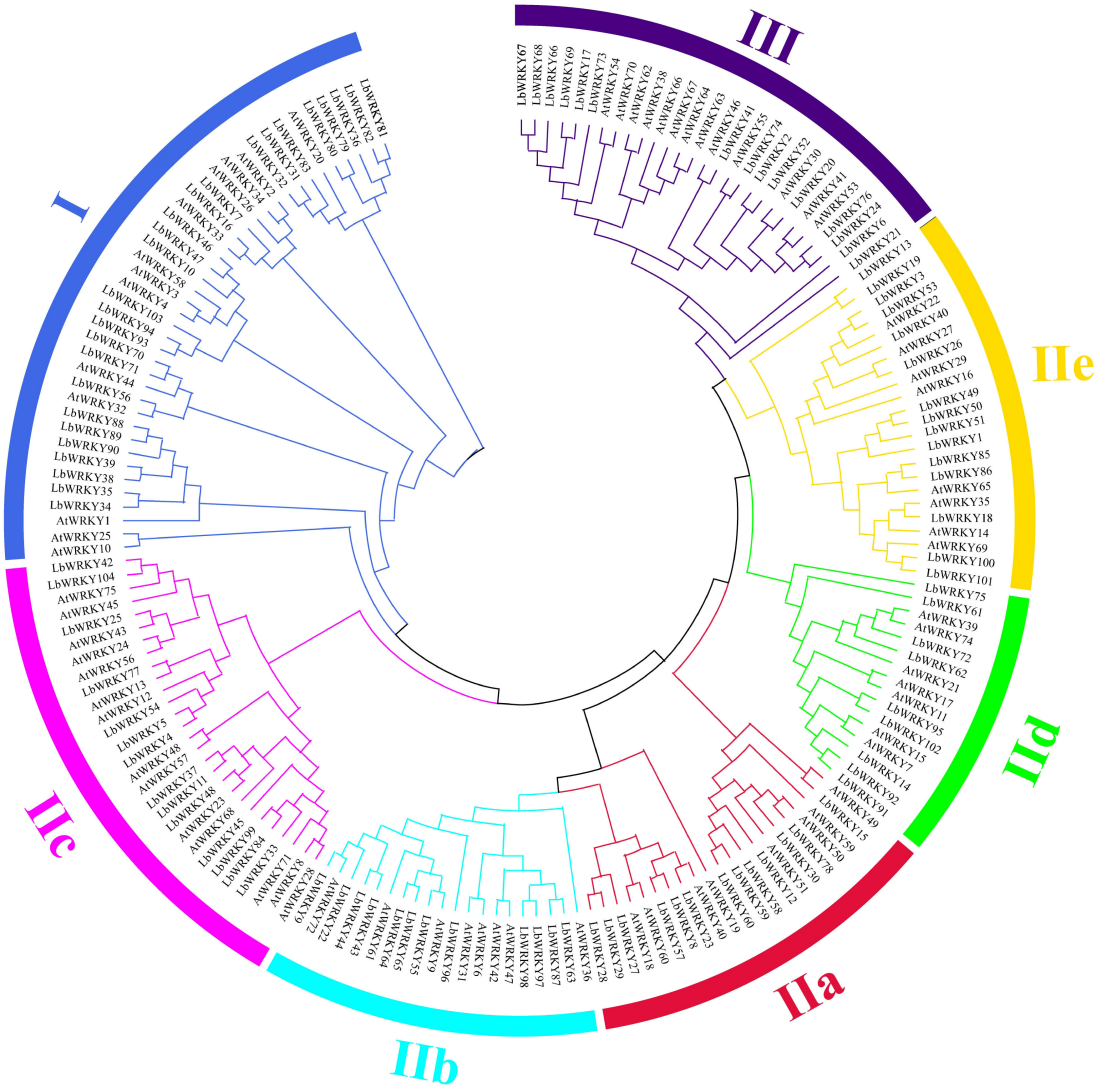
The phylogenetic tree of WRKY proteins from wolfberry and *Arabidopsis thaliana*. These proteins were clustered in three main groups represented by different colors, with each subgroup marked outside the circle as I, II and III. Lb, *Lycium barbarum*; At, *Arabidopsis thaliana*.

To further understand the evolutionary relationship of WRKY gene in *L. barbarum*, a total of 175 WRKY genes, including 104 from wolfberry, 71 from in *Arabidopsis thaliana*, were used to construct an unrooted phylogenetic tree (**Figure 2**). The tree organized *WRKY* into three main groups. Group I included 39 members (26 *LbWRKY*) and (13 *AtWRKY* genes), and was characterized by two conserved WRKY domains (**Supplementary Figure S2**). Group II consisted 108 members, which were further subdivided into five subgroups: IIa (n = 21), IIb (n = 20), IIc (n = 28), IId (n = 16), and IIe (n = 23). Group III was the smallest containing 28 members: 15 WRKY genes from wolfberry and 13 from *Arabidopsis*. These results suggested that *L. barbarum* has a significantly greater number of WRKY genes compared to *A. thaliana*. This difference is likely due to the larger genome size of wolfberry, which has facilitated the expansion of WRKY genes during genome evolution.

### Gene structure and protein motif of *WRKYs* in *L. barbarum*

**Figure 3:**
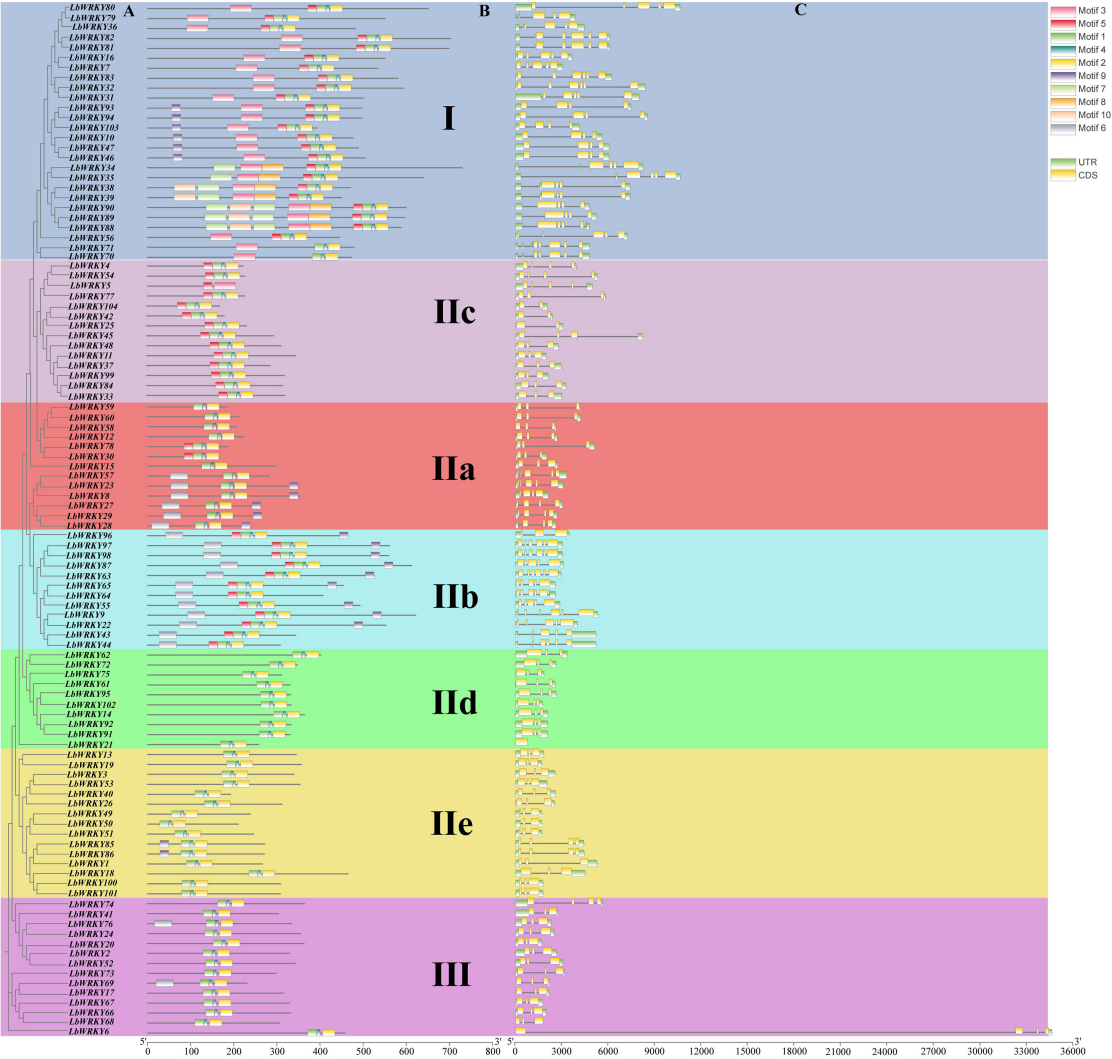
Phylogenetic clustering, conserved protein motifs and gene structure of *LbWRKY* genes. **(A)** A phylogenetic tree was constructed of *LbWRKYs*. Different groups and subgroups are displayed in different colors. **(B)** Use different color boxes to represent the motif. **(C)** Exon-intron structure of the *LbWRKYs*. Untranslated 5 ′ -and 3 ′ - regions, exons and introns Subs are represented by green box, yellow box and black line respectively.

To investigate of structural diversity *LbWRKY* genes, the analyses of exon-intron structures and conserved motifs were performed, followed by a phylogenetic analysis (**Figure 3**). The results indicated that the classification of 104 LbWRKY protein domains based on the multiple sequence alignment (**Figure 3A**) was consistent with the grouping in **Figure 1**. Members within the same subgroup exhibited similar gene structures and motif modules. Gene structure analysis showed that each *LbWRKY* consisted of a variable number of exons and introns (**Figure 3C**). The number of introns *LbWRKY* genes ranged from 0 to 5, with varying intron sizes. Group I featured 4–6 exons, *LbWRKY32* and *LbWRKY83* specifically contained 6 exons. Group II subgroups (IIa-e) contained 1–6 exons, with subgroup IId having the highest exon count: 4 members contained 6 exons, and 5 members had 5 exons. In Group III, only *LbWRKY6* and *LbWRKY74* contained 4 exons, while all other members had 3 exons. Apart from IId, most of the other groups contain 3 exons.

Using the MEME online tool, 10 conserved motifs in *LbWRKY* genes were predicted and named as motif 1 to motif 10 (**Supplementary Figure S3**). The results revealed that all members of the LbWRKY gene family contained the motif 1, 2 and 4. However, these motifs varied across the phylogenetic groups (**Figure 3B**). Motif 1and 3 contained the WRKYGQK sequence, proteins in Group I predominantly contained the motifs 1-5, while Group IId, IIe and III mainly contained motifs 1, 2 and 4, and Group IIc consisted of motif 1, 2, 4 and 5, except for LbWRKY5, which contained only two motifs, motifs 3 and 5. The similarity in gene structure, motif content and distribution patterns within each phylogenetic group indicates a shared evolutionary origin. Conversely, the uniqueness of motifs within phylogenetic groups reflects the functional differences among *LbWRKY* members.

### Collinearity and synteny analyses

**Figure 4:**
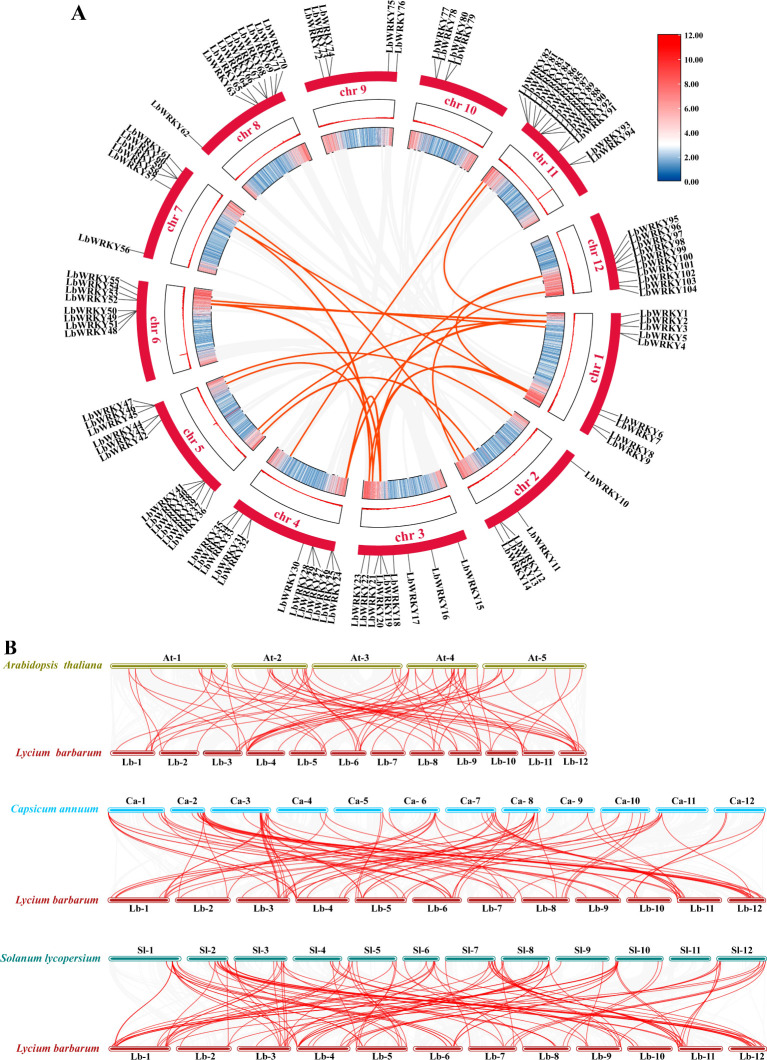
**(A)** The outermost circle represented the identified members of the WRKY gene family in *Lycium barbarum*, the red bars represent different chromosomes, and the inner colored bars represented the gene density on the chromosomes. **(B)** Colinearity analysis of wolfberry with *Arabidopsis*, pepper and tomato, with colored lines above and below each set collinearity represented chromosomes of different species.

Segmented, scattered or tandem gene replication events in the genome are regarded as a major driving of evolution (Panchy et al., 2016). Segmental duplication analysis of the 104 *LbWRKY* genes by TBtools and Circos identified 30 homologous sites and 22 pairs of segmental duplication events (**Figure 4A**; **Supplementary Table S3**). However, 19 *LbWRKY* genes lacated on chromosomes 8, 9 and 10 were derived from dispersed and tandem chromosomal distributions. The results of the Ka/Ks ratio analysis for homologous genes showed that 18 ratios were less than 1.0, showing that the *LbWRKY* gene family may have undergone strong purifing during evolution (**Supplementary Table S2**).

The collinearity of *L. barbarum WRKYs* with those of *A. thaliana*, *C. annuum* and *S. lycopersium* was investigated (**Figure 4B**), and there are 71, 98 and 117 pairs of colinear genes, respectively (**Supplementary Table S4**). A significant number of genes exhibited collinearity across three selected species, suggesting that these genes may play a critical role in the evolution of *LbWRKY* gene family.

### Cis-acting elements analysis of *LbWRKY* genes promotors

**Figure 5:**
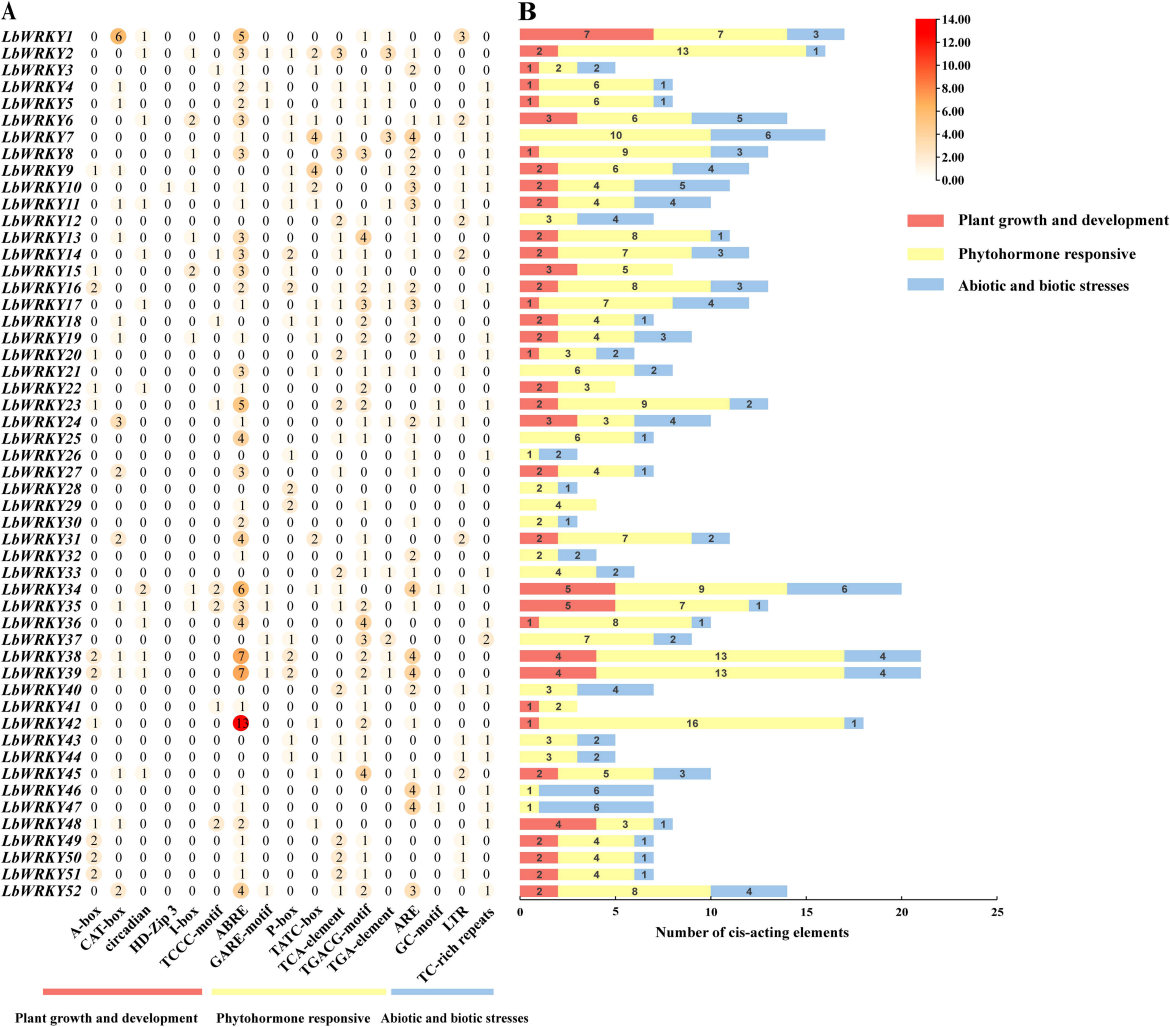
Analysis of cis-acting elements in promoter regions of *LbWRKY1-52*. **(A)** Three categories of cis-acting elements in the *LbWRKYs*. Different number and color of the circles representing the number of different cis elements. **(B)** The accumulation map of cis-acting elements in each *LbWRKY* gene. Different colors representing different categories of cis-acting elements.

Gene expression can be regulated by the binding of transcription factors (TF)to cis-acting elements (Wang et al., 2014), which is closely related to their adaptability to developmental and stress responses. The cis-acting elements of 104 *LbWRKY* genes were analyzed using PlantCARE. As shown in **Figure 5**, a total of 992 cis-acting elements were identified and classified into three basic categories: including plant growth and development, phytohormone responsive and abiotic and biotic stresses (**Supplementary Figure S4**, **Supplementary Table S5**). Among these, cis-acting elements related to phytohormone responsiveness accounted for 41.18% in second subgroup. *LbWRKY38* and *LbWRKY39* contained the biggest number of cis-elements (21), while *LbWRKY26*, *LbWRKY28*, *LbWRKY30* and *LbWRKY41* contained the lowest number (3). Phytohormone response elements such as ABRE 21.67% and TGACG-motif 11.59%, antioxidant response elements ARE 15.12% were widely distributed in the promoter regions of *LbWRKY* genes. However, only *LbWRKY10* and *LbWRKY80* contained cis-acting elements involved in plant growth and development, such as the HD-ZIP 3 element, which is associated with meristem formation, lateral organ morphogenesis, and vascular development.

### Expression profile of *LbWRKY* gene family in different developmental stages of *Lycium barbarum* and prediction of miRNA target and SSR

To elucidate the expression profiles of *LbWRKY* members in response to *Fusarium solani* infection during the development of *L. barbarum*, the expression patterns of 28 *LbWRKY* genes were analyzed using the Genevestigator platform (**Supplementary Figure S7**). The results indicated that the *LbWRKY* family was widely expressed across different tissues and developmental stages of *L. barbarum*. Notably, LbWRKY7, LbWRKY8 and LbWRKY14 exhibited significantly higher expression levels, suggesting their crucial roles in the growth and development. Then, the 3D structural models of LbWRKY7, LbWRKY8 and LbWRKY14 are predicted by AlphaFold tool. The results showed that the protein tertiary structure contain alpha helix and random coil (**Supplementary Figure S8**).

MicroRNAs (miRNAs) plays a significant role in plant gene regulation (Millar, 2020). In this study, tomato miRNA was used to predict miRNA targets of LbWRKY genes in *Lycium barbarum*. A total of 32 different miRNA (**Supplementary Table S6**) were predicted, of which *LbWRKY72*, *LbWRKY100* and *LbWRKY101* contained the largest number of miRNA targets (n=9). Using Trf software to analyze the SSR of *LbWRKY* family, there are 5 kinds of SSR sequences, which are CAA, AAACAA, CCA, GCA and TAA, among which CCA sequence has the most replication times, with a total of 21 times. In addition, the sequence similarity between the repeats of *LbWRKY62* and *LbWRKY18* was the highest, which was 93%.

### Analysis expression pattern of *LbWRKY* genes after inoculation with *F. solani*

**Figure 6:**
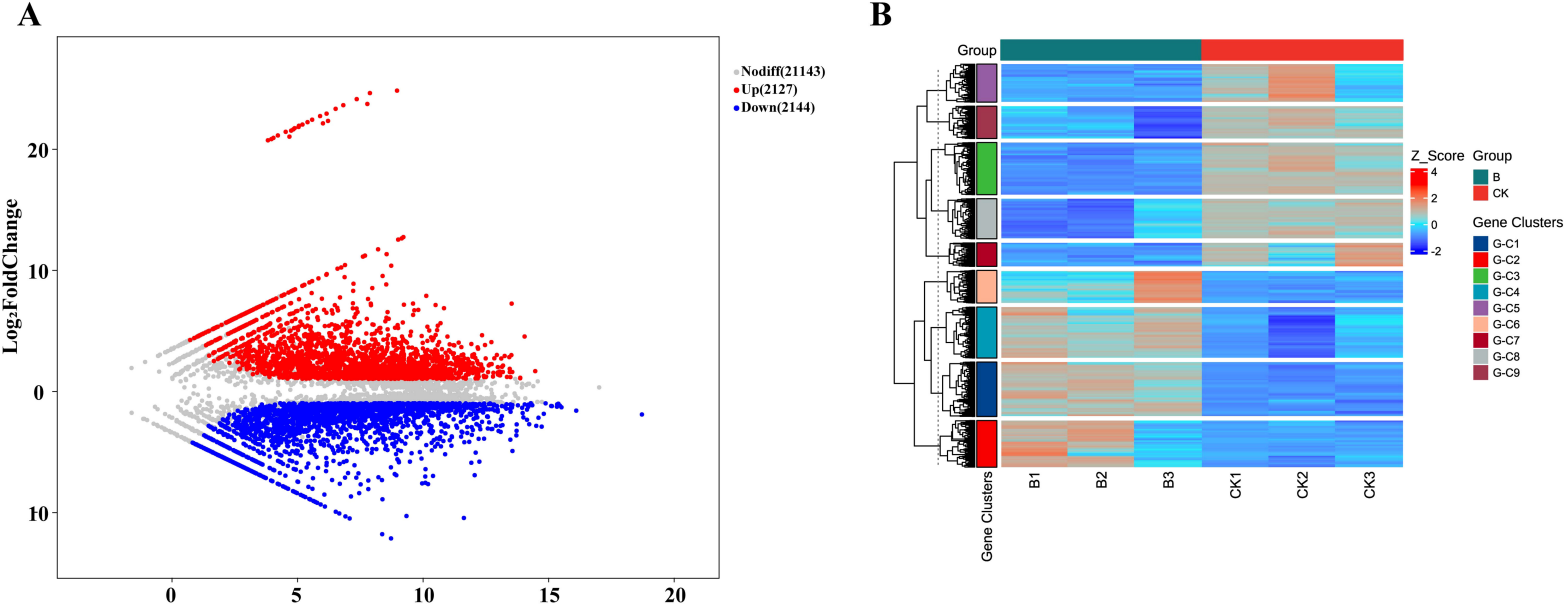
DEGs expression pattern of Wolfberry under root rot pathogen stress. **(A)** MA plot (M-versus-A plot) of DEGs. Color indicates whether the gene is up-regulated, down-regulated or non-significantly differentially expressed. **(B)** Cluster analysis heat map of DEGs. Gene clusters, Gene clustering classification tags, genes with similar expression patterns are clustered into a cluster (G-C1, C2 etc).

Previous research has demonstrated that WRKY transcription are widely involved in plant disease resistance pathways (Rushton et al., 2010). We analyzed the transcriptome of *L. barbarum* inoculated with *F. solani*, a total of 4271 genes were differentially expressed, of which 2144 were up-regulated and 2127 down-regulated (**Figure 6A**). These differential genes were clustered into seven modules according to their similar expression patterns (**Figure 6B**). These results indicated that pathogen invasion triggers significant changes in gene expression in *L. barbarum*.

KEGG pathway analysis of the transcriptome data highlighted several enriched pathways, including plant hormone signal transduction, phenylpropanoid biosynthesis and flavonoid biosynthesis, suggesting that the synthesis of stimulant metabolites in wolfberry plays an important role in the resistance to *F. solani* (**Supplementary Figure S5**). In addition, statistical analysis of TF families showed significant changes in 38 TFs, among which most members of the ERF, MYB, bHLH families involved in the resistance response (**Supplementary Figure S6**). Notably, the expression of 28 *WRKY* members was up-regulated, making them the second most up-regulated TF family after to ERF.

### Correlation analysis of DEGs between *LbWRKYs* and involved in flavonoid biosynthesis and phenylpropanoid biosynthesis

**Figure 7:**
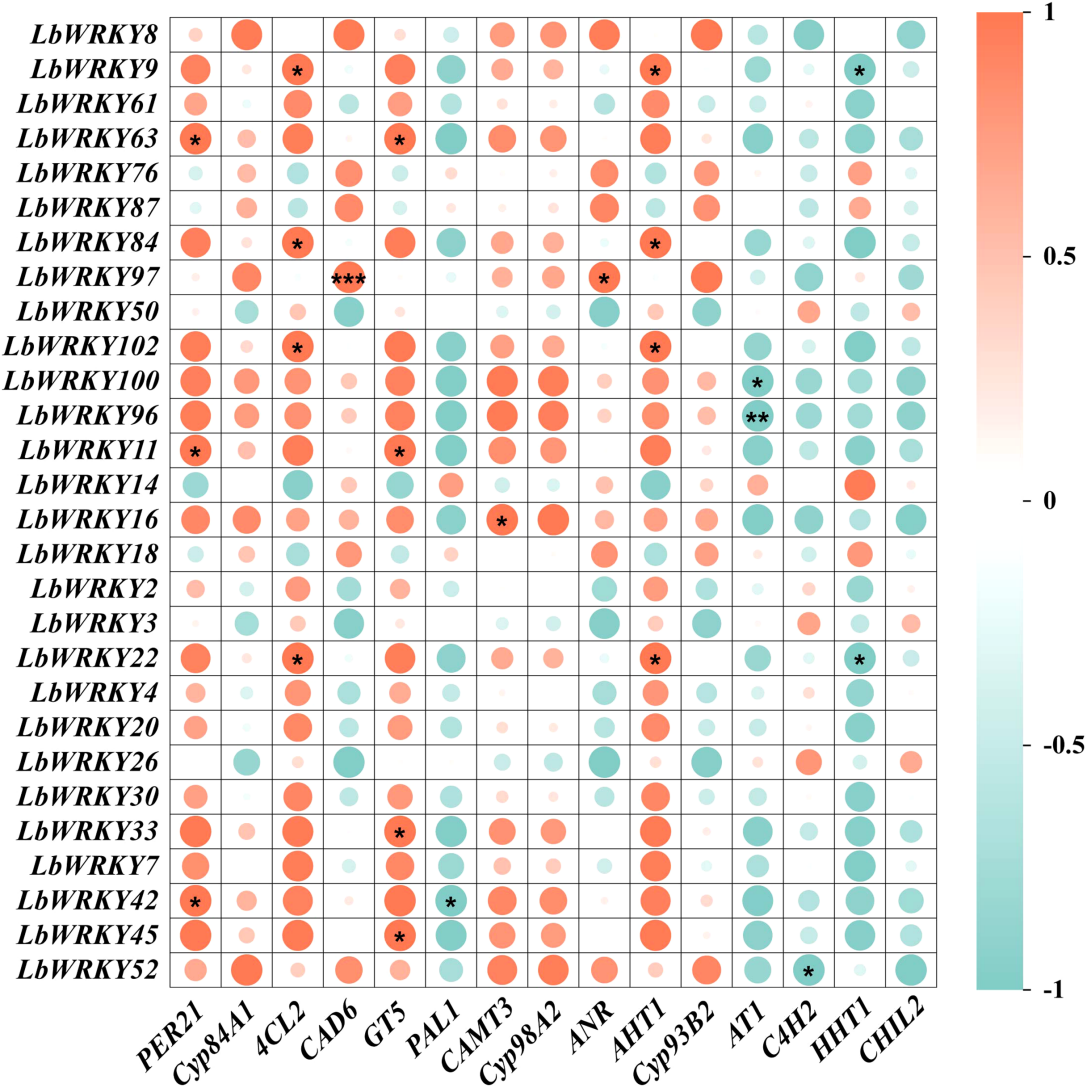
The correlation analysis of DEGs between *LbWRKYs* and involved in flavonoid biosynthesis and phenylpropanoid biosynthesis. The depth of color represents the strength of correlation. *P < 0.05, **P < 0.01, ***P < 0.001.

As a key components of plant defense, secondary metabolites play a crucial role in plant disease resistance (Hou et al., 2024). It has been demonstrated that WRKY TF regulate the synthesis of secondary metabolites (Lu et al., 2023; Zhao et al., 2024). To elucidate the role of WRKY family members in regulating flavonoids and phenylpropane synthesis in *L. barbarum* for enhanced resistance to root rot, we analyzed the correlation between WRKYs and synthesis-related genes in response to *F. solani* (**Supplementary Table S7**, **Supplementary Table S8**). The expression of 28 *LbWRKY* genes changed significantly, with some showing strong correlations with the key genes of flavonoid and phenylpropanoid synthesis (**Figure 7**). *LbWRKY63* displayed a significant positive correlation with *PER21* and *GT5*, while *LbWRKY84* and *LbWRKY22* were positively correlated with *4CL2* and *AHT1*. A highly significant positive correlation was observed between *LbWRKY97* and *CAD6*. conversely, most relationships between *AT1*, *C4H2* and *HHT1* genes and these *LbWRKYs* were negatively correlated. These results showed that LbWRKY transcription factors regulate the synthesis of flavonoids and phenylpropanoids in *L. barbarum*, thereby contributing to resistance against *F. solani*.

### Prediction regulatory network of LbWRKYs and synthesis-related genes of flavonoids and phenylpropane

**Figure 8:**
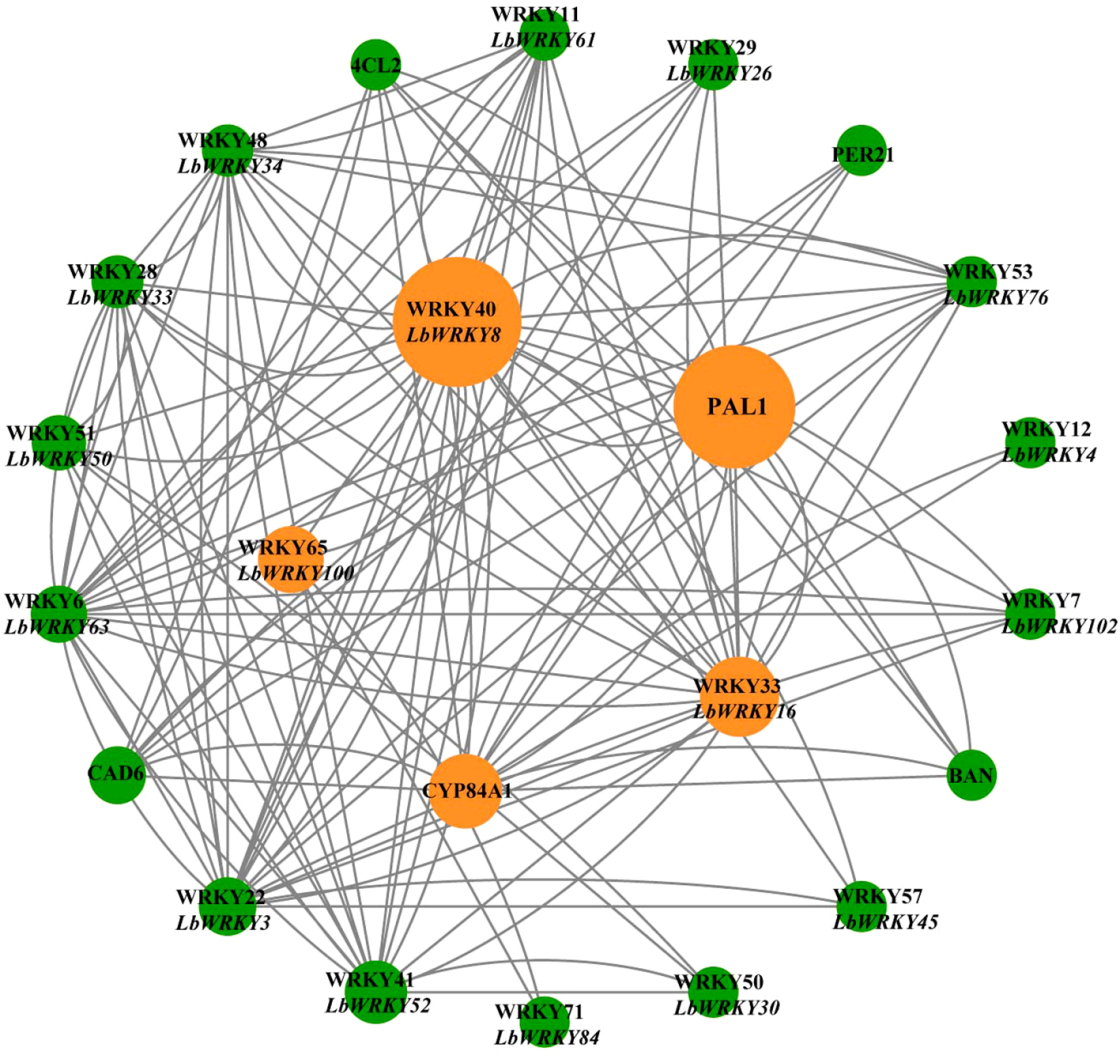
Protein regulatory network of LbWRKYs and synthesis-related genes of flavonoids and phenylpropane. Different color circles represented *WRKYs* members and flavonoid phenylpropanoid synthesis-related genes in response to *F. solani*, and the grey lines represent possible regulatory relationships. The inner circle represents the genes with more regulatory relationships. The size of the circle represents the degree of connection. If the circle was larger, the degree of connection was higher.

Utilizing the STRING database, the regulatory network of *LbWRKY* genes and synthesis-related genes in response to *F. solani* was predicted, with *Solanum lycopersicum* serving as a reference species (**Figure 8**). This network analysis enabled us to infer the potential functions of *LbWRKY* genes in the synthesis of secondary metabolites. *LbWRKY8*, *LbWRKY100*, *LbWRKY16*, *LbWRKY3*, *LbWRKY33* and *LbWRKY63* exhibited a strong correlation to synthesis of secondary metabolites, particularly phenylpropanoid biosynthesis, as deduced from their roles in *S. lcopersicum*. These findings suggested that *LbWRKY* genes may be involved in regulating plant growth and development and stress resistance process.

### Expression profile of *LbWRKY* genes in response to *Fusarium solani*

**Figure 9:**
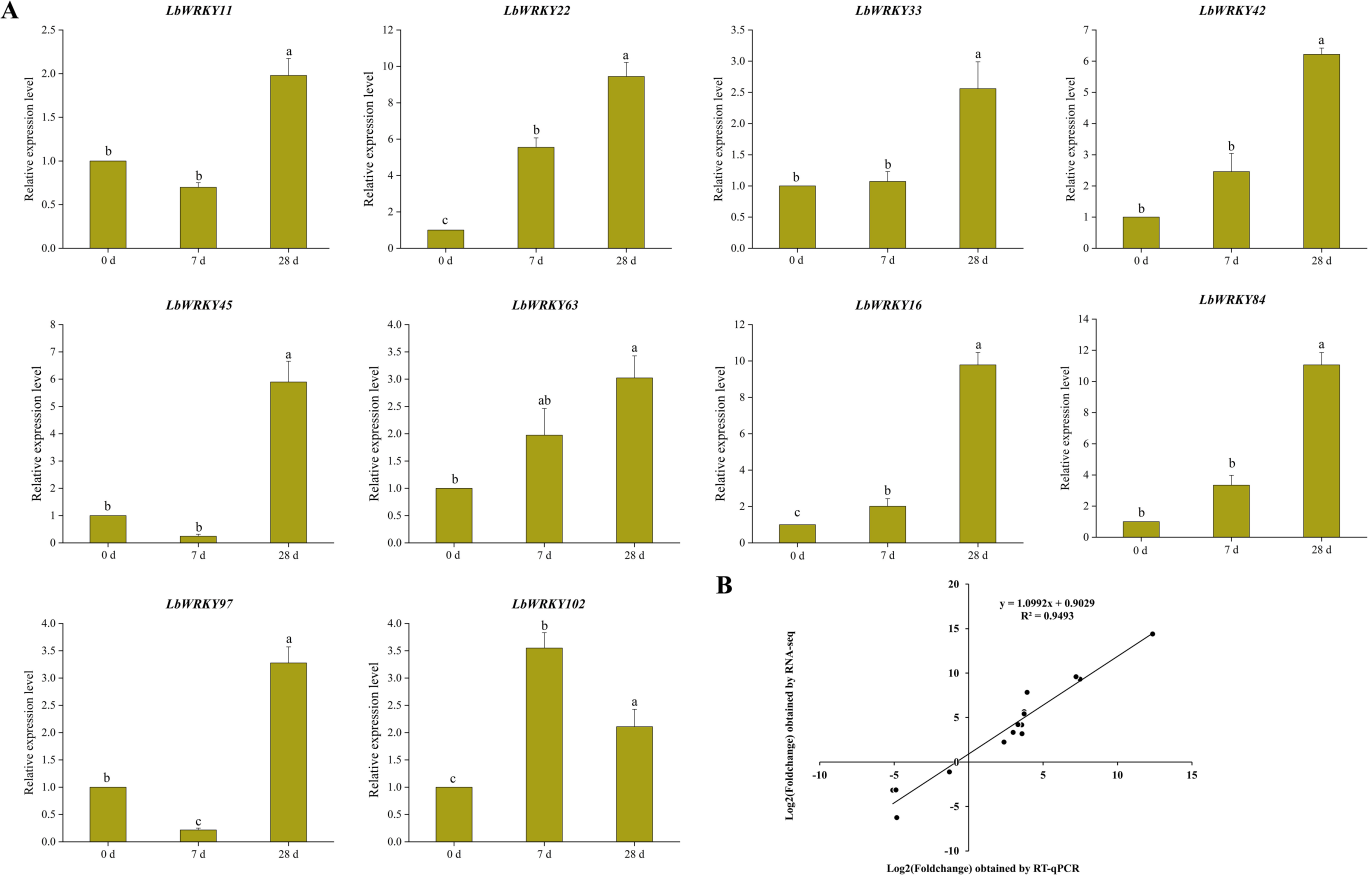
Response of relative expression of *LbWRKY* genes to *F*. *solani* and verification of RNA-seq data. **(A)** The expression levels of *LbWRKY* genes at 0 dpi, 7 dpi and 28 dpi, Lowercase letters indicate significant differences among different inoculation time (*p*-value<0.05). **(B)** Standard curve of log2Foldchange between RNA-Seq and qRT-PCR values. Lowercase letters indicate significant differences (P<0.05).

In order to clarify the expression characteristics of *LbWRKY* genes in *L. barbarum* during the incubation and disease development stages, we analyzed relative gene expression levels at 7 dpi and 28 dpi following inoculation with *F. solani*. The results showed that the expression of *LbWRKY* genes increased significantly after inoculation with *F. solani*, with some genes, including *LbWRKY22*, *LbWRKY33*, *LbWRKY42*, *LbWRKY16* and *LbWRKY84*, showing continuous upregulation as the infection progressed (**Figure 9A**). It speculated that *LbWRKYs* play crucial roles in the disease resistance. Additionally, a standard curve was established to compare the expression levels of 12 related genes obtained through RNA-seq and RT-qPCR, yielding a correlation coefficient of 0.9493. The high correlation indicated strong consistency between the RNA-seq and RT-qPCR results (**Figure 9B**).

## Discussion

WRKY TFs are one of the most prominent families in higher plants, playing crucial roles in plant growth and responses to both biotic and abiotic stresses (Ahmed et al., 2022). With the advancements in genomics and transgenic technology, the functions of *WRKY* gene family members in plant adaptation to environmental stress have become perspicuity. There were 76, 73, 81 and 116 *WRKY* gene family members in *Cucurbita pepo* (Chen et al., 2024), *Capsicum chinense* (Zhang et al., 2023), *solanum lycopersicum* (Khan et al., 2023) and *Gossypium* (Dou et al., 2014), they have been shown to play an active role in the biological process. In the present study, a total of 104 *LbWRKY* genes were identified in the *L. bararum* genome, 28 of which were responsive to the infection of *F. solani*.

The *WRKY* gene family is categorized into three groups based on highly conserved zinc finger motifs and WRKY domains (Eulgem et al., 2000). The *LbWRKY* gene family was divided into group I, II (IIa, IIb, IIc, IId, IIe), and III, with the proportion of WRKY members in each group being 25%, 60.6%, and14.4%, respectively. Notably, members of group II constitute more than half of the total. However, in *Arabidopsis*, group II members account for only 40% (Wang et al., 2011). It is speculated that members of group II underwent more gene duplication processes in wolfberry. Three variations of heptapeptide sequence, WRKYGKK, WRKYGMK and WRKYGQN were identified in *LbWRKY* genes, which may be associated with the amplification of *WRKY* gene in *L. barbarum*. In pepper (*Capsicum annuum*), the replacement of Q with M in the conserved WRKYGQK in *CaWRKY27b* prevents its binding to W-boxes. Instead, *CaWRKY27b* interacts with *CaWRKY40* in the nucleus, positively regulates the tolerance of pepper to high temperature and the resistance to *Ralstonia solanacearum* (Yang et al., 2022). These variations are hypothesized to influence the expression of stress-responsive genes targeted by *LbWRKY* transcription factors. Further investigation is needed to elucidate the interaction mechanism of LbWRKY proteins in *L. barbarum*.

Conserved motif analysis provides essential information for understanding the evolution of gene family (Hu et al., 2015). The analysis of protein motifs showed that nearly all members of the group II contained motifs 1,2 and 4, but motif 5 was specific to subgroups IIc and IIb. In addition, there were motifs 6 and 9 were present in certain members of IIa and all members of IIb. The similarity in the distribution of conserved motifs among members of the same group indicated functional similarities. The promoter regions of *WRKY* gene contains stress-related cis-acting elements (Hayashi et al., 2013). We identified 992 cis-acting elements related to plant growth and development, plant hormone response, and abiotic and biotic stress. Notably, plant hormone-related elements accounted for 41.18%, indicating that the *LbWRKY* genes are involved in various plant hormones regulation pathways.

Plant-specific WRKY TFs play diverse roles in various plant processes, including growth, development, and stress signaling through both autonomic and cross-regulation of numerous genes (Bakshi and Oelmüller, 2014). Previous researches have demonstrated that WRKY TFs participate in the response to abiotic stress. For instance, sugarcane transcription factor ScWRKY4 has been shown to negatively regulate resistance to pathogen infection (Wang et al., 2024a). In *Panax notoginseng*, PnWRKY9 positively regulates the resistance to root rot caused by *F. solani* (Zheng et al., 2022). In *S. lycopersicum*, *SlWRKY30* and *SlWRKY81* work synergistically against modulate immunity to *Ralstonia solanacearum* (Dang et al., 2023). In this study, 28 LbWRKY members responded to *F. solani*, including *LbWRKY2*, *LbWRKY8*, *LbWRKY33*, *LbWRKY30* and *LbWRKY50*, among others. This finding is consistent with previous studies, suggesting a close association between the *LbWRKY* gene family and the resistance of wolfberry to pathogens. In addition, transcriptome analysis of *L. barbarum* after inoculation with *F. solani* revealed differential expression of genes related to flavonoid and phenylpropanoid synthesis pathways. Notably, there were strong correlations between *WRKY* members and genes related to flavonoid and phenylpropanoid synthesis in response to *F. solani*. These results provide further evidence that *WRKY* members regulate the synthesis of flavonoids and phenylpropanoids thereby enhancing disease resistance (Wen et al., 2021; Ma F et al., 2023).

Protein-protein interactions (PPI) are essential for the regulation of plant growth, and the stimulation of transcription factors to proteins activates various stress pathways in plants (Braun et al., 2013; Francois et al., 2020). The PPIs of *LbWRKY* genes and synthesis-related genes were analyzed using String database, revealing strong correlations. For example, WRKY40 had high degree value of 211.02. Among these, the homologue of *LbWRKY8* in *S. lycopersicum* was identified as *WRKY40* (Solyc03g116890), which acts as positive regulator in effector-triggered immunity against the bacterial pathogen *Pseudomonas syringae* DC3000 (Moritz et al., 2013). In other Solanaceae plants, *WRKY40* plays a positive role in disease resistance. For instance, *CaWRKY40* mediates autoregulation during the response to *R*. *solanacearum* in pepper (Liu et al., 2020). In addition, *CaWRKY40* binds to the W4-box element of the *ChiIV3* promoter region, activating transcription and enhancing resistance to *R. solanacearum* (Liu et al., 2019). In summary, it speculated that LbWRKY gene family may play an active role in resistance to root rot. Interestingly, a significant correlation was observed between *LbWRKY* genes and genes involved in flavonoid and phenylpropanoid biosynthesis, including PAL, 4CL2, CAD6 and PER21. The heterologous expression of *CcWRKY25* from *Capsicum chinense* in *Arabidopsis* promoted the expression of *PAL*, *4CL1*, *4CL2*, *4CL3*, *CCR* and *CCoAOMT*, leading to the accumulation of lignin and flavonoids (Zhang et al., 2023). Similarly, in *Ocimum sanctum*, overexpression of *OscWRKY1* positively regulated genes in the phenylpropanoid pathway and enhances resistance to pathogens (Joshi et al., 2022). These researches further illustrate that the *LbWRKY* gene family in *L. barbarum* regulates resistance to *F. solani* by modulating flavonoid and phenylpropanoid synthesis pathways.

## Conclusion

In the current study, the WRKY gene family in the whole genome of *Lycium barbarum* was identified, comprising a total of 104 members. The bioinformatics identification and characteristics of the *LbWRKY* genes were conducted, and collinearity analysis with the WRKY gene families of other Solanaceae plants showed their conserved evolutionary nature. Transcriptome data revealed 28 LbWRKY genes responding to *Fusarium solani*, and played a key role in regulating flavonoid and phenylpropanoid synthesis pathways in *Lycium barbarum*. These results provide valuable insights into the functional roles of *LbWRKY* genes in root rot resistance in *L. bararum* and lay a foundation for the breeding disease-resistant cultivars of wolfberry.

## Data Availability

The original contribution presented in this study is included in the article/**Supplementary Material**, and the raw RNA-seq data are freely available in the NCBI database under accession no. PRJNA1260582.
