# Supplementary figures and images for: Genome-wide identification of WRKY gene family and expression analysis of key WRKY genes in response to Fusarium solani infection in Lycium barbarum
Source: Front Plant Sci. 2025 May 19;16:1543373. doi: 10.3389/fpls.2025.1543373 (PMC12127359; doi:10.3389/fpls.2025.1543373)

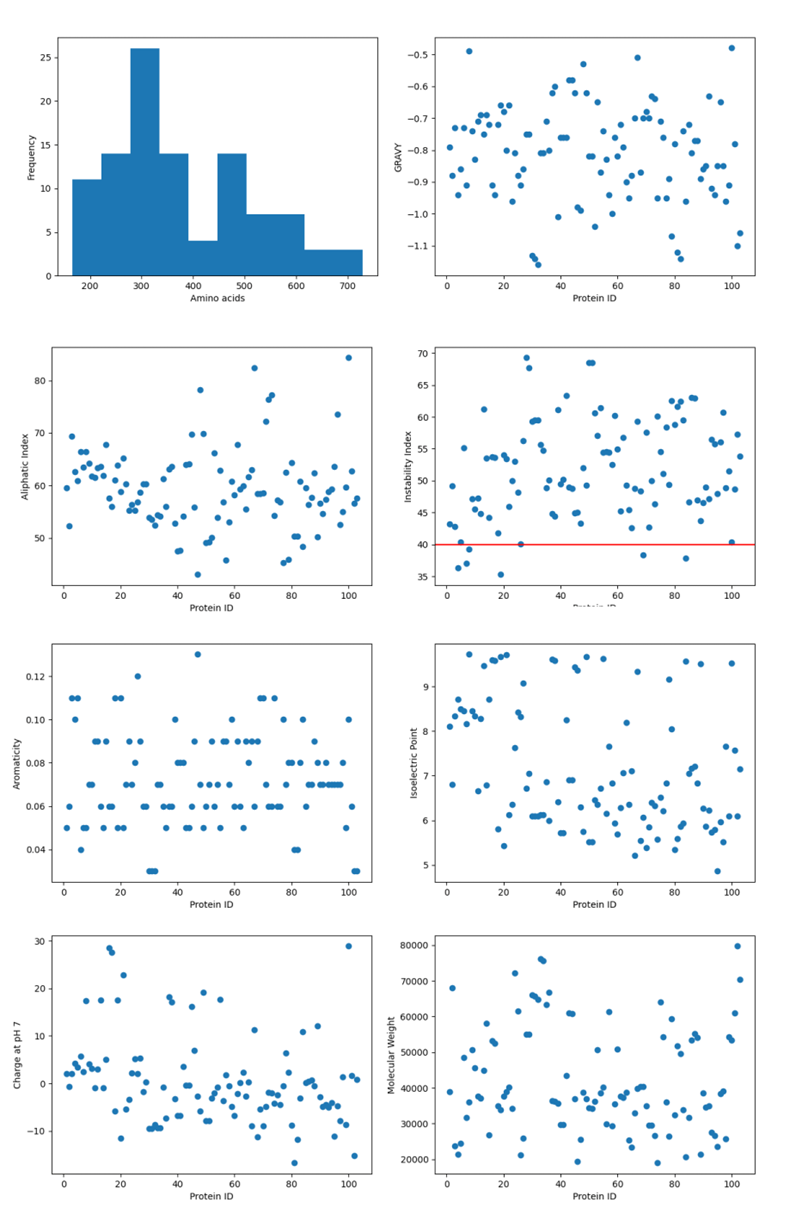

Supplement: Supplementary file 1 [file Image1.png]

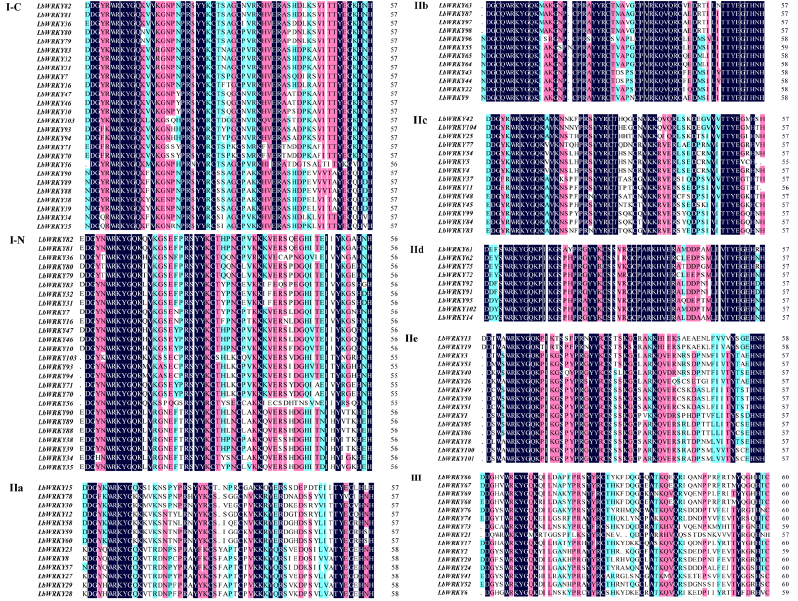

Supplement: Supplementary file 2 [file Image2.png]

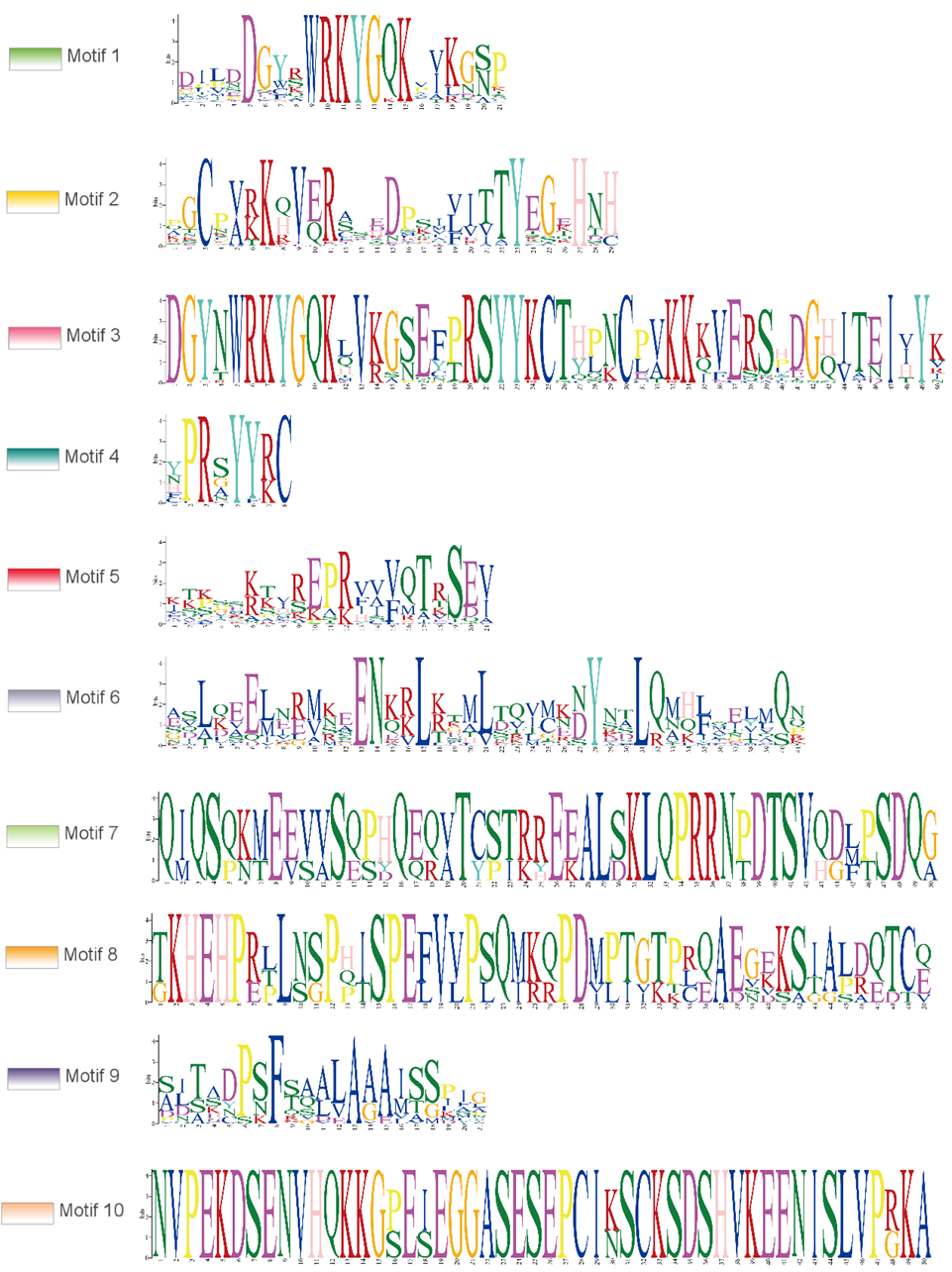

Supplement: Supplementary file 3 [file Image3.png]

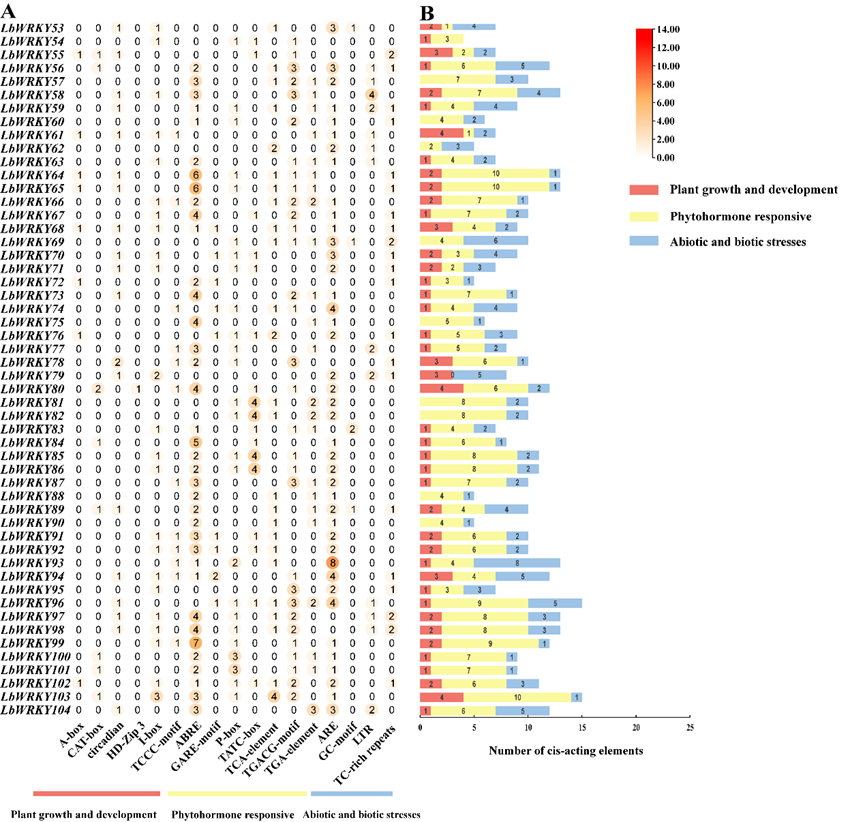

Supplement: Supplementary file 4 [file Image4.png]

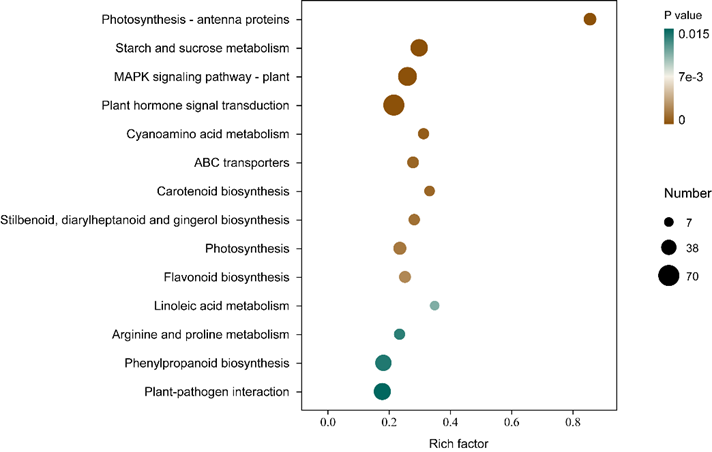

Supplement: Supplementary file 5 [file Image5.png]

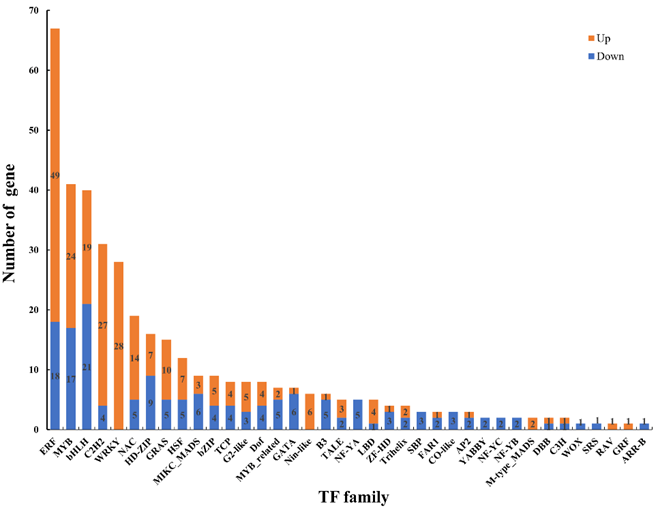

Supplement: Supplementary file 6 [file Image6.png]

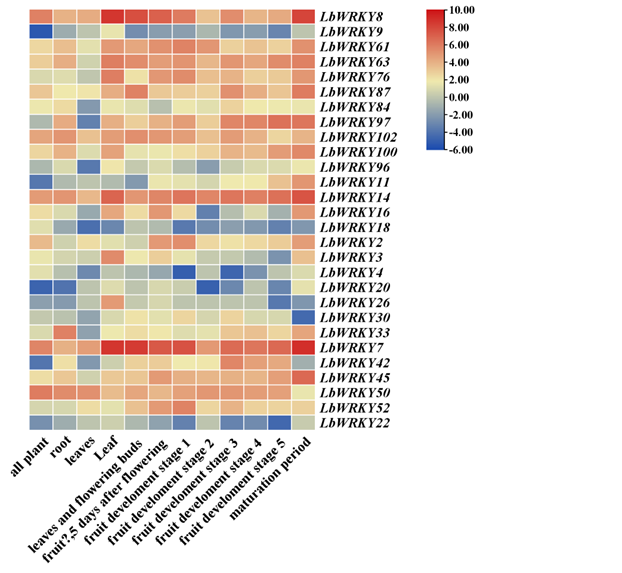

Supplement: Supplementary file 7 [file Image7.png]

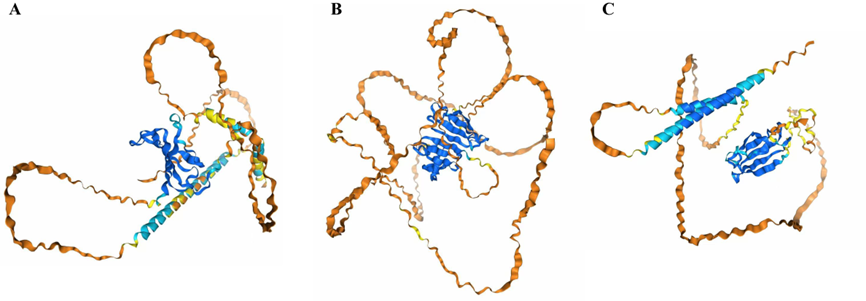

Supplement: Supplementary file 8 [file Image8.png]
